# Efficacy and safety of the enhanced monofocal intraocular lens in glaucoma of varying severity

**DOI:** 10.1038/s41598-025-87282-3

**Published:** 2025-02-08

**Authors:** Heesuk Kim, Junyeong Ahn, Minju Seo, Hyoung Won Bae, Chan Yun Kim, Wungrak Choi

**Affiliations:** 1 Institute of Vision Research, Department of Ophthalmology, Yonsei University College of Medicine, 50-1, Yonsei-ro, Seodaemun-gu, Seoul, 03722 Republic of Korea; 2 Yonsei University College of Medicine, Seoul, Republic of Korea

**Keywords:** Enhanced monofocal intraocular lenses, Glaucoma severity, Visual outcomes, Visual field test, Retinal nerve fiber layer thickness, Best-corrected visual acuity, Medical research, Outcomes research

## Abstract

This study evaluates the safety and visual outcomes of enhanced monofocal intraocular lenses compared to standard monofocal intraocular lenses in patients with varying severities of glaucoma. Utilizing data from surgeries performed in 2021, the study involved patients aged 40 and older with visually significant cataracts and diagnosed glaucoma or glaucoma suspects. The findings indicate that both enhanced and standard monofocal lenses lead to significant improvements in best-corrected visual acuity, visual field index, mean deviation, and retinal nerve fiber layer thickness postoperatively. No significant differences were observed between the two groups in the extent of these improvements, suggesting that enhanced monofocal lenses may offer a viable alternative for patients with glaucoma, providing significant visual benefits and potentially better intermediate vision while preserving overall visual function.

**Supplementary Information:**

The online version contains supplementary material available at 10.1038/s41598-025-87282-3.

## Introduction

Glaucoma is a progressive optic neuropathy characterized by visual field (VF) loss and optic nerve damage^1^. It often leads to central vision defects, further compromising the patient’s vision and quality of life (QOL)^1,2^. Globally, glaucoma is a leading cause of irreversible blindness and its prevalence increases with age^3,4^. Cataract, the leading cause of visual impairment worldwide is also highly prevalent in older adults^5^, with prevalence increasing significantly with age^6^. Glaucoma management is challenging in older adults when cataract surgery is required. As both conditions often coexist, their combined impact on vision can significantly diminish the QOL.

Although multifocal intraocular lenses (IOLs) provide multiple focal points, they may reduce contrast sensitivity and induce photic phenomena, such as halos and glare, diminishing their suitability for patients with glaucoma^7–9^. Multifocal IOLs provide significantly lower mean deviation (MD) values compared with monofocal IOLs^10,11^. This is particularly concerning in patients with advanced glaucoma, where visual function is already compromised. As glaucoma progresses, patients experience significant reductions in contrast sensitivity and overall visual function, including central vision defects^12–14^, limiting the use of multifocal IOLs^15,16^.

However, previous studies have shown that nondiffractive, wavefront-shaping, extended depth-of-focus IOLs overcome the aforementioned disadvantages of multifocal IOLs^17–19^, even in patients with mild glaucoma^20^. Recently developed enhanced monofocal IOLs improve the depth of focus while minimizing visual disturbances, such as glare and contrast sensitivity loss, making them a better option for patients with glaucoma^21–23^. They provide better intermediate vision, higher satisfaction, and lower spectacle-dependence compared with standard monofocal IOLs, without worsening other visual outcomes in patients with early glaucoma^24^.

Despite these advancements, research on the safety and efficacy of enhanced monofocal IOLs in patients with advanced glaucoma is limited. Therefore, we aimed to evaluate the safety and visual outcomes of enhanced monofocal IOLs in patients with glaucoma, contributing to more informed IOL selection in this population.

## Materials and methods

### Study design

This single-center, retrospective, comparative study was conducted at the Yonsei University College of Medicine, Severance Eye Hospital in Seoul, South Korea, using data collected from surgeries performed in 2021. The study protocol was reviewed and approved by the Institutional Review Board (IRB file number: 2-2023-0260) of Gangnam Severance Hospital, Seoul, Republic of Korea. All procedures adhered to the tenets of Declaration of Helsinki. Due to the retrospective nature of the study, the IRB waived the need for obtaining informed consent. Furthermore, this study contained no personal information, and the data were analyzed anonymously. We reviewed the medical records of patients who underwent cataract surgery with enhanced or standard monofocal IOL insertion.

### Participants

We sought to recruit participants aged ≥ 40 years with visually significant cataracts who had been previously diagnosed with glaucoma or glaucoma suspect (GS). Patients who underwent cataract surgery and were eligible for follow-up of 3 months or more were enrolled. Exclusion criteria included corneal abnormalities, such as corneal opacity or pterygium; retinal diseases, including age-related macular degeneration or branch retinal vein occlusion; severe cataracts that prevented accurate glaucoma testing; active uveitis; combined surgery for glaucoma and cataracts, except iStent surgery; traumatic or complicated cataracts; or any other ocular pathology that may limit visual function.

All participants were categorized by glaucoma type. GS was defined as changes in the optic nerve head, including generalized or focal increases in the optic cup size and increases > 0.6 in the cup-disc ratio, narrowing or notching of the neural rim, optic nerve hemorrhage, and cup-disc ratio asymmetry > 0.2 between the right and left eyes. Glaucoma was diagnosed based on glaucomatous optic disc changes or nerve fiber layer defects with the following corresponding VF defect criteria: (1) cluster of three or more points with a p-value < 5%, of which one had a p-value < 1%; (2) pattern standard deviation value < 5%; and (3) a result of “outside normal limits” on the Glaucoma Hemifield Test in a 24 − 2 VF. Primary open-angle glaucoma (POAG) is characterized by an open anterior chamber angle on gonioscopy. Primary angle-closure glaucoma (PACG) is characterized by a closed angle/occludable angle > 180° on gonioscopy before laser peripheral iridotomy.

Further, all cases of glaucoma were controlled with medication, maintaining intraocular pressure (IOP) < 21 mmHg or at a safe level to prevent further optic nerve damage, with no changes in the glaucoma medication regimen and no VF deterioration over the past year.

Only one eye per patient was included in this analysis, selecting the first eye treated to minimize individual variability. Overall, this study included 296 eyes from 296 patients; enhanced monofocal and standard monofocal IOLs were implanted in 156 and 140 eyes, respectively.

### Preoperative examination

Complete ophthalmic examinations were conducted within 3 months post-surgery. The primary outcomes included the best-corrected visual acuity (BCVA), VF index (VFI), MD, retinal nerve fiber layer thickness (RNFLT), and IOP. The BCVA was measured using an electronic target system (CCP-3100; Huvitz, Gunpo, Republic of Korea) under standardized testing conditions. Distance correction was applied using values obtained from an autorefractor, followed by subjective refraction to ensure optimal visual correction and all VA results were converted into the logarithm of the minimum angle of resolution for statistical analysis. Data on the patient’s basic characteristics were also obtained.

Cirrus FastTrac eye-tracking technology with software version 6.0 (Cirrus OCT, Carl Zeiss Meditec, Inc., Jena, Germany) was employed to measure the retinal nerve fiber layer (RNFL). Automated segmentation was also performed. Standard automated perimetry was performed using the Swedish Interactive Threshold Algorithm standard 24 − 2 program of the Humphrey Field Analyzer II (Carl Zeiss Meditec, Inc., Jena, Germany). Only reliable VF testing results (fixation loss < 20%, false-positive errors < 15%, and false-negative errors < 15%) were used. The IOP was assessed using a Goldmann applanation tonometer.

### Surgical technique

All cataract surgeries were performed by three expert glaucoma surgeons using standard phacoemulsification techniques with in-the-bag IOL implantation. Phacoemulsification was performed using the same device (Centurion; Alcon Laboratories Inc., Geneva, Switzerland). Using the clear-cornea technique, 2.8-mm incisions were made and sealed with stromal hydration. The postoperative refractive goal (diopter) was determined using the Barrett Universal II formula. Postoperative medications included topical steroids, antibiotics, and non-steroidal anti-inflammatory eye drops.

### IOLs

Two types of IOLs from the same manufacturer (Johnson & Johnson Vision Care, Inc., Jacksonville, Florida, USA) were evaluated. The Tecnis^®^-1 model ZCB00 (ZCB IOL) is a single piece, 6.0-mm ultraviolet light-absorbing hydrophobic acrylic monofocal IOL. It has a biconvex anterior aspheric surface and a square optic edge with a posterior spherical surface. The Tecnis^®^ Eyhance, model ICB00 (ICB IOL) is a recently developed enhanced monofocal IOL that uses the same platform and biconvex design as the ZCB IOL. It comprises a higher-order aspheric anterior surface and provides an enhanced intermediate VA. Moreover, it facilitates a continuous and faster power increase from the periphery to the center than the ZCB IOL, which provides enhanced intermediate VA. The choice of IOL was based on patient preferences after explaining the characteristics of each IOL, regardless of their visual field test results or glaucoma severity.

### Postoperative examination

Complete ophthalmic examinations were conducted at least 3 months post-surgery to allow sufficient recovery time and minimize the potential impact of postoperative dry eye syndrome on the assessment of visual outcomes. The evaluations included BCVA, VFI, MD, RNFL, and IOP measurements. The observation period was limited to 1 year to avoid the influence of glaucoma progression or other diseases. Patients with increased glaucoma medications, uncontrolled glaucoma progression, or new conditions that could confound the results were excluded to minimize variability and ensure a reliable comparison of visual outcomes between the different intraocular lens types.

### Statistical analysis

Statistical analyses were performed using SAS software (version 9.4; SAS Institute, Cary, North Carolina, USA). The Kolmogorov–Smirnov and Shapiro–Wilk tests were used to determine data normality, guiding the use of parametric or non-parametric methods. Baseline characteristics are expressed as the mean ± standard deviation. The independent t-test, paired t-test, Wilcoxon signed-rank, Mann-Whitney U, and chi-squared tests were used to verify differences between the enhanced and standard monofocal IOL groups. Statistical significance was set at *p* < 0.05.

### Meeting presentation

This abstract (ID: 30078660) has been selected for poster presentation at the 2024 American Academy of Ophthalmology (AAO) Annual Meeting.

## Results

### Baseline characteristics

Overall, 296 eyes underwent enhanced monofocal (*n* = 156) or standard monofocal (*n* = 140) IOL implantation. Demographic and preoperative characteristics of the groups are summarized in Table 1. The mean age was significantly different between the enhanced and standard monofocal IOL groups (66.08 ± 9.22 vs. 68.31 ± 8.46 years; *p* = 0.030). The sex distribution also differed significantly (*p* = 0.043), with a higher percentage of men in the standard monofocal IOL group (50.71%) than in the other group (39.74%). Laterality (*p* = 0.318), preoperative BCVA (*p* = 0.142), RNFLT (*p* = 0.088), or baseline IOP (*p* = 0.117) were not significantly different. However, the preoperative VFI (*p* = 0.025) and MD (*p* = 0.015) differed significantly between the groups.

**Table 1:** Demographic and preoperative characteristics of the two IOL groups.

Parameter	All Eyes (*n* = 296 eyes)	Enhanced monofocal IOL (*n* = 156 eyes)	Standard monofocal IOL (*n* = 140 eyes)	*p*-value
Age (years)	67.14 ± 8.92	66.08 ± 9.22	68.31 ± 8.46	0.030*
Sex (M/F) (patients)	133 (44.93%)/163 (55.07%)	62 (39.74%)/94 (60.26%)	71 (50.71%)/69 (49.29%)	0.043*
Laterality (R/L) (eyes)	190(64.19%)/106(35.81%)	105(67.31%)/51(32.69%)	85(60.71%)/55(39.29)	0.318
Duration (days)				
Between pre-operative examination and surgery	110.28 ± 19.84	107.13 ± 21.37	113.72 ± 18.10	0.773
Between surgery and post-operative exam	229.35 ± 20.96	220.43 ± 18.18	239.02 ± 23.64	0.448
Baseline visual indices				
Pre-operative BCVA (logMAR)	0.48 ± 0.47	0.44 ± 0.45	0.52 ± 0.49	0.142
Pre-operative VFI (%)	78.51 ± 26.71	81.82 ± 24.86	74.83 ± 28.28	0.025*
Pre-operative MD (dB)	-8.81 ± 8.29	-7.69 ± 7.94	-10.05 ± 8.53	0.015*
Pre-operative RNFL thickness (µm)	76.40 ± 17.21	78.03 ± 16.20	74.58 ± 18.17	0.088
Baseline IOP (mmHg)	15.49 ± 6.46	14.91 ± 3.84	16.13 ± 8.45	0.117
*Glaucoma type*				0.028*
Open-angle glaucoma	159 (53.72%)	74 (47.44%)	85 (60.71%)	0.027*
Glaucoma suspect^a^	81 (27.36%)	53 (33.97%)	28 (20.00%)	0.009*
Angle-closure glaucoma	44 (14.86%)	21 (13.46%)	23 (16.43%)	0.515
Secondary glaucoma	12 (4.05%)	8 (5.13%)	4 (2.86%)	0.386

Continuous variables were analyzed using independent t-test. Categorical variables are presented using descriptive statistics as numbers (%) using the χ2 test.

Glaucoma type distribution includes comparisons of the proportions of glaucoma suspect, primary open-angle glaucoma, primary angle-closure glaucoma, and secondary glaucoma between the two groups. Post-hoc comparisons for specific types are provided

^a^Glaucoma suspect was defined as changes in the optic nerve head, including generalized or focal increases in the optic cup size and increases > 0.6 in the cup-disc ratio; narrowing or notching of the neural rim; optic nerve hemorrhage; and a cup-disc ratio asymmetry > 0.2 between the two eyes. *p-value < 0.05

BCVA, best-corrected visual acuity; IOL, intraocular lens; VFI, visual field index; MD, mean deviation; RNFL, radiating nerve fiber layer; IOP, intraocular pressure; logMAR, logarithm of the minimum angle of resolution

Differences in glaucoma type were also observed between the two groups (*p* = 0.028). The Post-hoc comparisons showed that enhanced monofocal IOL group included a higher proportion of glaucoma suspect patients (33.97% vs. 20.00%; *p* = 0.009), while the standard monofocal IOL group had a higher proportion of patients with primary open-angle glaucoma (60.71% vs. 47.44%; *p* = 0.027). There were no significant differences in the proportions of primary angle-closure glaucoma (*p* = 0.515) or secondary glaucoma (*p* = 0.386) between the two groups.

### Visual outcomes by IOL type

The visual outcomes according to IOL type are summarized in Table 2. Both groups exhibited significant postoperative improvements in the BCVA, MD, and RNFLT scores and IOP (all *p* < 0.05), with no significant inter-group differences. Despite these improvements, the magnitude of these changes did not differ significantly between the groups (BCVA, *p* = 0.905; MD, *p* = 0.463; RNFLT, *p* = 0.632; IOP, *p* = 0.827). The VFI showed a trend towards improvement, albeit without statistical significance in either group (enhanced: *p* = 0.186, standard: *p* = 0.813), while the magnitude of change also lacked significance (*p* = 0.519).

**Table 2:** Comparison of visual outcomes according to IOL type.

Visual outcomes	Enhanced monofocal IOL (*n* = 156 eyes)				Standard monofocal IOL (*n* = 144 eyes)				*p*-value^2^
	Pre-operative	Post-operative	Difference	*p*-value^1^	Pre-operative	Post-operative	Difference	*p*-value^1^	
BCVA (logMAR)	0.43 ± 0.44	0.06 ± 0.24	0.37 ± 0.40	< 0.001*	0.52 ± 0.49	0.15 ± 0.37	0.36 ± 0.36	< 0.001*	0.905
VFI (%)	81.86 ± 24.93	83.23 ± 25.48	1.37 ± 12.87	0.186	75.34 ± 27.73	75.65 ± 28.54	0.31 ± 15.37	0.813	0.519
MD (dB)	-7.64 ± 7.94	-5.89 ± 8.46	1.75 ± 4.38	< 0.001*	-9.91 ± 8.39	-8.54 ± 9.28	1.37 ± 4.63	0.001*	0.463
RNFLT (µm)	78.03 ± 16.20	84.16 ± 15.44	6.13 ± 10.18	< 0.001*	74.58 ± 18.17	80.07 ± 14.98	5.49 ± 12.70	< 0.001*	0.632
IOP	14.91 ± 3.84	13.59 ± 5.62	-1.32 ± 5.72	0.005*	14.14 ± 8.36	12.65 ± 3.04	-1.49 ± 7.57	< 0.001*	0.827

BCVA, best-corrected visual acuity; IOL, intraocular lens; VFI, visual field index; MD, mean deviation; RNFLT, radiating nerve fiber layer thickness; IOP, intraocular pressure, logMAR: logarithm of the minimum angle of resolution

p-value^1^ indicates the difference within each group; p-value^2^ represents the comparison of the differences between two groups

*p < 0.05

### Visual outcomes by glaucoma type

Table 3 details visual outcomes by glaucoma type. In patients with POAG, both IOL groups demonstrated significant improvements in BCVA, RNFLT, and IOP (all *p* < 0.05), without significant inter-group differences (BCVA, *p* = 0.384; RNFLT, *p* = 0.101; IOP, *p* = 0.073). Similar trends were observed for GS, with significant improvements in BCVA, MD, and RNFLT (all *p* < 0.05), and no significant inter-group differences (BCVA, *p* = 0.979; MD, *p* = 0.881; RNFLT, *p* = 0.354). In patients with PACG, both IOL groups showed significant improvements in the BCVA and MD (all *p* < 0.05), albeit without significant inter-group differences (BCVA, *p* = 0.305; MD, *p* = 0.411). For both GS and PACG groups, postoperative IOP reductions in the Enhanced Monofocal IOL group were not statistically significant. However, the differences in IOP changes between the Enhanced and Standard Monofocal IOL groups were also not statistically significant (GS, *p* = 0.637; PACG, *p* = 0.168). The VFI did not improve significantly in any glaucoma type, except for GS in the enhanced monofocal IOL group. Overall, the differences in all parameters between the two groups were not significant.

**Table 3:** Comparison of visual outcomes by glaucoma type.

Outcomes	Pre-operative	Post-operative	Difference	*p*-value^1^	Pre-operative	Post-operative	Difference	*p*-value^1^	*p*-value^2^
POAG	Enhanced (*n* = 74)				Standard (*n* = 85)				
BCVA	0.41 ± 0.44	0.07 ± 0.26	-0.34 ± 0.36	< 0.001*	0.53 ± 0.46	0.14 ± 0.28	-0.39 ± 0.36	< 0.001*	0.384
VFI	77.11 ± 25.57	76.46 ± 28.03	-0.65 ± 13.56	0.682	70.48 ± 27.69	71.61 ± 28.96	1.13 ± 17.50	0.553	0.480
MD	-9.11 ± 8.23	-8.22 ± 9.05	0.89 ± 4.41	0.086	-11.15 ± 8.39	-9.85 ± 9.26	1.30 ± 5.12	0.022*	0.596
RNFLT	72.26 ± 14.75	79.18 ± 12.52	6.92 ± 6.99	< 0.001*	71.45 ± 13.52	76.47 ± 13.33	5.02 ± 7.41	< 0.001*	0.101
IOP	14.85 ± 3.88	13.23 ± 2.98	-1.62 ± 3.31	< 0.001*	15.30 ± 6.59	12.31 ± 3.00	-2.99 ± 6.04	< 0.001*	0.073
GS	Enhanced (*n* = 53)				Standard (*n* = 28)				
BCVA	0.46 ± 0.48	0.04 ± 0.24	-0.41 ± 0.45	< 0.001*	0.44 ± 0.46	0.11 ± 0.45	-0.33 ± 0.26	< 0.001*	0.979^†^
VFI	92.43 ± 13.98	96.60 ± 4.82	4.17 ± 13.43	0.042*	88.21 ± 24.99	90.89 ± 19.39	2.68 ± 18.86	0.579^‡^	0.192^†^
MD	-4.27 ± 4.75	-1.08 ± 2.50	3.20 ± 4.64	< 0.001*	-6.25 ± 7.55	-2.91 ± 6.66	3.33 ± 5.65	0.002^‡^*	0.881^†^
RNFLT	87.17 ± 12.68	92.23 ± 15.65	4.73 ± 10.93	0.003*	82.37 ± 14.86	91.46 ± 12.23	8.89 ± 14.43	0.001^‡^*	0.354^†^
IOP	14.68 ± 3.40	13.92 ± 8.69	-0.76 ± 8.52	0.517	14.27 ± 2.70	12.90 ± 2.91	-1.37 ± 2.83	0.014^‡^*	0.637^†^
PACG	Enhanced (*n* = 21)				Standard (*n* = 23)				
BCVA	0.50 ± 0.51	0.01 ± 0.03	-0.49 ± 0.52	< 0.001^‡^*	0.47 ± 0.52	0.19 ± 0.51	-0.28 ± 0.32	< 0.001^‡^*	0.305^†^
VFI	78.48 ± 28.10	81.33 ± 25.46	2.86 ± 9.10	0.087^‡^	79.70 ± 26.48	81.17 ± 24.88	1.48 ± 8.91	0.434^‡^	0.252^†^
MD	-9.07 ± 8.61	-7.22 ± 8.76	1.85 ± 3.80	0.022^‡^*	-9.07 ± 8.21	-7.70 ± 8.16	1.37 ± 2.77	0.018^‡^*	0.411^†^
RNFLT	80.43 ± 18.06	88.10 ± 15.20	7.67 ± 16.97	0.064^‡^	83.39 ± 23.93	82.04 ± 17.42	-1.35 ± 17.01	0.876^‡^	0.126^†^
IOP	14.51 ± 4.72	13.37 ± 2.11	-1.15 ± 4.07	0.210	18.38 ± 11.86	13.26 ± 3.32	-5.13 ± 10.31	0.020^‡^*	0.168^†^

POAG, primary open-angle glaucoma; GS, glaucoma suspect, PACG, primary closed-angle glaucoma; BCVA, best-corrected visual acuity; VFI, visual field index; MD, mean deviation; RNFLT, radiating nerve fiber layer thickness; IOP, intraocular pressure.

p-value^1^ represents the difference within each group; p-value^2^ indicates the comparison of the differences between two groups.

*p < 0.05; ^†^Mann-Whitney test; ^‡^Wilcoxon signed-rank test were applied for non-parametric data.

### Visual outcomes by glaucoma severity

Additional analyses were conducted to determine whether the visual outcomes differed based on the severity of glaucoma. From amongst the available VF grading systems^25^, the severity was categorized based on the MD values using the Hodapp, Parrish, and Anderson classification^26^ into early (MD ≥ -6 dB), moderate (-6 dB > MD ≥ 12 dB), severe (MD < -12 dB) disease (Table 4). In patients with early glaucoma, both IOL groups showed significant improvements in the BCVA, MD, and RNFLT (all *p* < 0.05), with no significant inter-group differences (BCVA, *p* = 0.356; MD, *p* = 0.810; RNFLT, *p* = 0.523). The VFI showed an improving trend, albeit without statistical significance, in both groups (enhanced, *p* = 0.231; standard, *p* = 0.791; between-group, *p* = 0.677). Similar results were observed for the moderate and severe glaucoma types, with significant improvements in the BCVA and RNFLT (all *p* < 0.05) and no significant inter-group differences (moderate: BCVA, *p* = 0.882; VFI, *p* = 0.647; MD, *p* = 0.753; RNFLT, *p* = 0.054; severe: BCVA, *p* = 0.133; VFI, *p* = 0.679; MD, *p* = 0.582; RNFLT, *p* = 0.370). For IOP, significant reductions were observed in all severities in both IOL groups except for early glaucoma in the enhanced monofocal IOL group (*p* = 0.192). No significant inter-group differences were observed across all severities (early, *p* = 0.577; moderate, *p* = 0.408; severe, *p* = 0.163).

**Table 4:** Comparison of visual outcomes by glaucoma severity.

Outcomes	Pre-operative	Post-operative	Difference	*p*-value^1^	Pre-operative	Post-operative	Difference	*p*-value^1^	Pre-operative	Post-operative	Difference	*p*-value^1^	*p*-value^2^
Early	Total (*n* = 153)				Enhanced (*n* = 88)				Standard (*n* = 65)				
BCVA	0.36 ± 0.34	0.02 ± 0.08	-0.34 ± 0.34	< 0.001*	0.37 ± 0.38	0.01 ± 0.03	-0.36 ± 0.38	< 0.001*	0.35 ± 0.28	0.05 ± 0.11	-0.31 ± 0.28	< 0.001*	0.356
VFI	95.21 ± 4.37	95.64 ± 4.96	0.43 ± 5.49	0.332	95.48 ± 3.65	96.07 ± 4.80	0.59 ± 4.59	0.231	94.85 ± 5.20	95.06 ± 5.14	0.22 ± 6.54	0.791	0.677
MD	-3.01 ± 1.88	-1.72 ± 2.53	1.29 ± 2.61	< 0.001*	-2.84 ± 2.03	-1.59 ± 2.56	1.25 ± 2.76	< 0.001*	-3.25 ± 1.64	-1.89 ± 2.50	1.35 ± 2.40	< 0.001*	0.810
RNFLT	82.88 ± 14.02	87.61 ± 13.71	4.73 ± 10.60	< 0.001*	83.42 ± 13.27	88.63 ± 14.52	5.20 ± 9.62	< 0.001*	82.15 ± 15.04	86.25 ± 12.52	4.09 ± 11.85	0.007*	0.523
IOP	14.54 ± 3.91	13.41 ± 5.47	-1.13 ± 5.78	0.016*	14.85 ± 3.59	13.93 ± 6.93	-0.92 ± 6.70	0.192	14.19 ± 4.30	12.70 ± 2.11	-1.41 ± 4.23	0.009*	0.577
Moderate	Total (*n* = 70)				Enhanced (*n* = 40)				Standard (*n* = 30)				
BCVA	0.54 ± 0.48	0.08 ± 0.22	-0.46 ± 0.44	< 0.001*	0.52 ± 0.46	0.06 ± 0.27	-0.46 ± 0.43	< 0.001*	0.55 ± 0.51	0.09 ± 0.15	-0.46 ± 0.46	< 0.001^‡^*	0.882^†^
VFI	84.04 ± 9.06	84.26 ± 15.30	0.21 ± 13.98	0.898	83.00 ± 8.92	85.08 ± 12.33	2.08 ± 8.73	0.141	85.43 ± 9.21	83.17 ± 18.71	-2.27 ± 18.74	0.991^‡^	0.647^†^
MD	-8.28 ± 1.83	-5.92 ± 4.90	2.37 ± 4.70	< 0.001*	-8.22 ± 1.81	-5.43 ± 4.30	2.79 ± 3.80	< 0.001*	-8.37 ± 1.87	-6.56 ± 5.61	1.80 ± 5.71	0.045^‡*^	0.753^†^
RNFLT	76.11 ± 14.40	82.51 ± 13.81	6.40 ± 11.03	< 0.001*	74.30 ± 14.58	82.28 ± 13.28	7.98 ± 10.60	< 0.001*	78.53 ± 14.05	82.83 ± 14.70	4.30 ± 11.41	0.010^‡^*	0.054^†^
IOP	14.63 ± 4.13	13.01 ± 2.70	-1.62 ± 3.29	< 0.001*	14.75 ± 4.25	13.45 ± 2.45	-1.30 ± 3.12	0.016*	14.48 ± 4.05	12.45 ± 2.93	-2.04 ± 3.52	0.004*	0.408^†^
Severe	Total (*n* = 73)				Enhanced (*n* = 28)				Standard (*n* = 45)				
BCVA	0.66 ± 0.62	0.30 ± 0.53	-0.36 ± 0.43	< 0.001*	0.54 ± 0.62	0.22 ± 0.42	-0.32 ± 0.49	< 0.001^‡^*	0.73 ± 0.61	0.34 ± 0.59	-0.39 ± 0.39	< 0.001*	0.133^†^
VFI	38.22 ± 23.17	42.11 ± 27.30	3.89 ± 25.74	0.387	37.21 ± 26.75	40.68 ± 31.54	3.46 ± 27.78	0.600^‡^	38.84 ± 20.93	43.00 ± 24.64	4.16 ± 24.72	0.265	0.679^†^
MD	-21.46 ± 6.11	-19.56 ± 8.32	1.90 ± 7.51	0.093	-22.19 ± 7.02	-19.91 ± 9.84	2.27 ± 8.12	0.232^‡^	-21.00 ± 5.51	-19.34 ± 7.33	1.66 ± 7.18	0.128	0.582^†^
RNFLT	62.72 ± 18.09	71.23 ± 14.37	7.62 ± 13.28	< 0.001*	66.00 ± 19.40	72.41 ± 15.06	6.41 ± 11.30	0.004^‡^*	60.70 ± 17.16	69.07 ± 12.42	8.36 ± 14.44	< 0.001*	0.370^†^
IOP	18.21 ± 10.42	12.66 ± 3.88	-5.55 ± 9.41	< 0.001*	15.29 ± 4.18	12.67 ± 3.57	-2.61 ± 4.78	0.007*	19.92 ± 12.46	12.65 ± 4.09	-7.27 ± 10.95	< 0.001*	0.163^†^

BCVA, best-corrected visual acuity; VFI, visual field index; MD, mean deviation; RNFTL, radiating nerve fiber layer thickness; IOP, intraocular pressure.

p-value^1^ represents the difference within each group; p-value^2^ indicates comparison of the differences between two groups.

*p < 0.05; ^†^Mann-Whitney test and ^‡^Wilcoxon signed-rank test were applied for non-parametric data.

### Visual outcomes by presence and severity of central VF defect

The VF defect patterns can vary even with the same degree of VF defect. Therefore, we analyzed whether there were differences in visual outcomes based on the presence and severity of a central VF defect. Central VF severity was determined by the number of significant points within the central 10°. According to the SCHEIE Visual Field Grading System, significance points were assigned based on the point values with specific rules for counting^27^ (Supplementary Material 1).

First, we examined whether there were differences in the visual outcomes based on the presence of central VF defects (Table 5). Both groups with and without central VF defects showed significant improvements in the BCVA, MD, RNFLT, and IOP postoperatively (all *p* < 0.05). However, the VFI did not improve significantly in either group (with central defect, *p* = 0.151; without central defect, *p* = 0.920). The magnitude of these improvements did not differ significantly between the two groups only except for IOP. (BCVA, *p* = 0.576; VFI, *p* = 0.202; MD, *p* = 0.481; RNFLT, *p* = 0.516; IOP, *p* < 0.05).

**Table 5:** Comparison of visual outcomes by the presence of a central visual field defect.

Visual outcomes	With central defect (*n* = 181 eyes)				Without central defect (*n* = 115 eyes)				*p*-value^2^
	Pre-op	Post-op	Difference	*p*-value^1^	Pre-op	Post-op	Difference	*p*-value^1^	
BCVA	0.51 ± 0.51	0.15 ± 0.39	-0.36 ± 0.39	< 0.001*	0.41 ± 0.39	0.03 ± 0.08	-0.39 ± 0.39	< 0.001*	0.576
VFI (%)	67.87 ± 29.33	69.85 ± 30.42	1.98 ± 18.47	0.151	95.26 ± 5.11	95.32 ± 6.52	0.06 ± 6.48	0.920	0.202
MD (dB)	-11.99 ± 9.08	-10.43 ± 9.78	1.56 ± 5.61	< 0.001*	-3.80 ± 2.54	-1.89 ± 3.13	1.91 ± 2.96	< 0.001*	0.481
RNFLT (µm)	71.61 ± 17.52	78.06 ± 15.56	6.17 ± 11.49	< 0.001*	83.86 ± 13.78	89.15 ± 12.42	5.29 ± 11.36	< 0.001*	0.516
IOP (mmHg)	16.16 ± 7.52	13.05 ± 5.42	-3.11 ± 8.14	< 0.001*	14.43 ± 4.02	13.26 ± 2.84	-1.16 ± 3.28	< 0.001*	< 0.001*

BCVA, best-corrected visual acuity; VFI, visual field index; MD, mean deviation; RNFLT, radiating nerve fiber layer thickness; IOP, intraocular pressure

p-value^1^ represents the difference within each group; p-value^2^ represents comparison of the differences between two groups

*p < 0.05

**Fig. 1:**
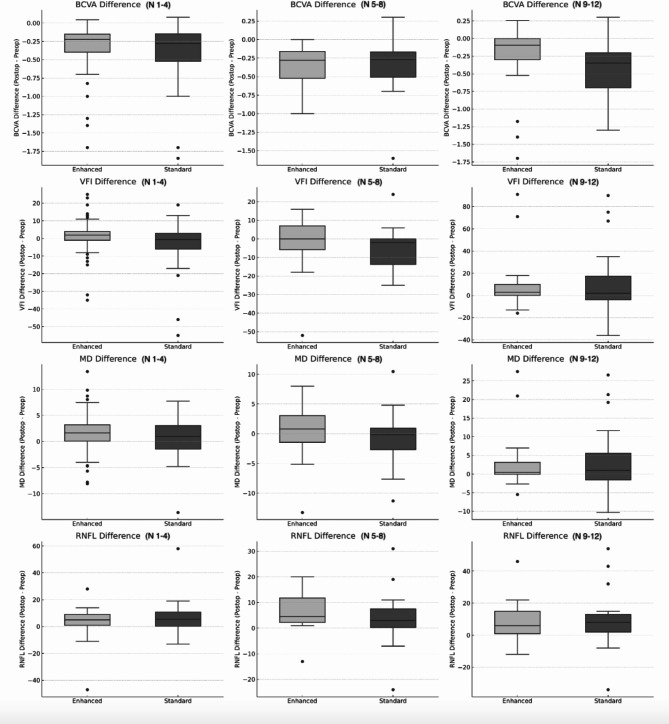
Comparison of visual outcomes by number of central field defects. ‘N 1–4’, ‘N 5–8’, and ‘N 9–12’ represent the number of significant points of central visual field defects. Enhanced and Standard: Comparison between enhanced and standard monofocal IOL groups. BCVA Difference: Difference in the best-corrected visual acuity (BCVA) before and after the operation across the groups. VFI Difference: Difference in the visual field index (VFI) before and after the operation across the groups. MD Difference: Difference in the mean deviation (MD) before and after the operation across the groups. RNFL Difference: Difference in the retinal nerve fiber layer (RNFL) thickness before and after the operation across the groups.

Next, we explored whether there were differences based on the severity of central VF defects between the two IOL types (Table 6; Fig. 1). Specifically, patients were divided into three groups based on the number of central VF defects (N 1–4:1 ≤ *n* ≤ 4; N 5–8:5 ≤ *n* ≤ 9; N 9–12:9 ≤ *n* ≤ 12). Irrespective of the severity of the central field defect, the BCVA and RNFLT increased significantly after surgery in all cases (all *p* < 0.05). Except for only one MD value, the VFI and MD did not show significant changes before and after surgery. Across all levels of central field defect severity, no significant differences were observed in the magnitude of improvement between the two IOL groups for any visual outcome except for IOP in N 5–8 (N 1–4, BCVA: *p* = 0.513, VFI: *p* = 0.083, MD: *p* = 0.312, RNFLT: *p* = 0.613, IOP: *p* = 0.361; N 5–8, BCVA: *p* = 0.904, VFI: *p* = 0.220, MD: *p* = 0.190, RNFLT: *p* = 0.181, IOP: *p* = 0.019; N 9–12, BCVA: *p* = 0.089, VFI: *p* = 0.933, MD: *p* = 0.950, RNFLT: *p* = 0.796, IOP: *p* = 0.522).

**Table 6:** Comparison of visual outcomes based on the extent of the central field defect.

Visual outcomes	Enhanced monofocal IOL				Standard monofocal IOL				*p*-value^2^
*N* 1–4	*n* = 58 eyes				*n* = 34 eyes				
	Pre-operative	Post-operative	Difference	*p*-value^1^	Pre-operative	Post-operative	Difference	*p*-value^1^	
BCVA (logMAR)	0.35 ± 0.35	0.02 ± 0.07	-0.32 ± 0.34	< 0.001*	0.47 ± 0.44	0.07 ± 0.13	-0.40 ± 0.44	< 0.001^‡^*	0.513^†^
VFI (%)	87.59 ± 10.01	88.83 ± 15.12	1.24 ± 9.96	0.347	90.24 ± 9.04	86.68 ± 17.92	3.56 ± 14.45	0.537^‡^	0.083^†^
MD (dB)	-5.91 ± 4.32	-4.24 ± 5.02	1.67 ± 4.00	0.002*	-5.84 ± 4.40	-5.03 ± 5.64	0.81 ± 4.14	0.118^‡^	0.312^†^
RNFLT (µm)	78.88 ± 15.36	83.19 ± 16.38	4.32 ± 9.78	0.002*	76.09 ± 15.76	82.53 ± 14.36	6.44 ± 11.24	< 0.001^‡^*	0.613^†^
IOP (mmHg)	14.98 ± 2.90	14.50 ± 8.24	-0.48 ± 8.08	0.652	15.13 ± 5.65	12.30 ± 2.93	-2.83 ± 5.17	< 0.001^‡^*	0.361^†^

*N* 5–8	*n* = 22 eyes				*n* = 18 eyes				*p*-value^2^
	Pre-operative	Post-operative	Difference	*p*-value^1^	Pre-operative	Post-operative	Difference	*p*-value^1^	
BCVA (logMAR)	0.47 ± 0.41	0.10 ± 0.36	-0.36 ± 0.29	< 0.001^‡^*	0.54 ± 0.49	0.20 ± 0.35	-0.34 ± 0.40	0.001^‡^*	0.904^†^
VFI (%)	71.36 ± 13.88	69.86 ± 22.14	-1.50 ± 14.59	0.793^‡^	61.28 ± 14.56	56.44 ± 19.49	-4.83 ± 12.46	0.103^‡^	0.220^†^
MD (dB)	-11.10 ± 4.81	-10.41 ± 7.38	0.68 ± 4.46	0.223^‡^	-13.95 ± 4.75	-14.61 ± 6.65	-0.66 ± 4.81	0.528^‡^	0.190^†^
RNFLT (µm)	72.14 ± 12.53	78.50 ± 11.56	6.36 ± 7.22	< 0.001^‡^*	71.00 ± 16.89	74.89 ± 13.11	3.89 ± 11.04	0.039^‡^*	0.181^†^
IOP (mmHg)	13.31 ± 3.18	12.37 ± 2.43	-0.93 ± 3.24	0.050	16.25 ± 6.80	12.55 ± 2.47	-3.70 ± 5.74	< 0.001	0.019^†^*

*N* 9–12	*n* = 17 eyes				*n* = 32 eyes				*p*-value^2^
	Pre-operative	Post-operative	Difference	*p*-value^1^	Pre-operative	Post-operative	Difference	*p*-value^1^	
BCVA (logMAR)	0.62 ± 0.71	0.31 ± 0.52	-0.31 ± 0.56	0.025^‡^*	0.83 ± 0.65	0.40 ± 0.65	-0.42 ± 0.40	< 0.001^‡^*	0.089^†^
VFI (%)	24.29 ± 27.36	35.41 ± 35.59	11.12 ± 28.07	0.132^‡^	32.84 ± 21.72	43.41 ± 30.91	10.56 ± 27.35	0.064^‡^	0.933^†^
MD (dB)	-24.90 ± 8.83	-21.55 ± 11.26	3.36 ± 8.41	0.118^‡^	-22.19 ± 6.60	-19.13 ± 9.45	3.05 ± 8.04	0.106^‡^	0.950^†^
RNFLT (µm)	62.76 ± 20.31	71.29 ± 16.38	8.53 ± 13.21	0.014^‡^*	58.16 ± 16.19	68.28 ± 12.19	9.19 ± 15.64	< 0.001^‡^*	0.796^†^
IOP (mmHg)	16.22 ± 3.92	12.45 ± 3.56	-3.77 ± 4.70	0.005^‡^*	21.26 ± 14.33	12.53 ± 4.37	-8.73 ± 12.43	< 0.001^‡^*	0.522^†^

BCVA, best-corrected visual acuity; logMAR, logarithm of the minimum angle of resolution; VFI, visual field index; MD, mean deviation; RNFLT, radiating nerve fiber layer thickness; IOP, intraocular pressure

p-value^1^ represents the difference within each group; p-value^2^ indicates comparison of the differences between two groups

*p < 0.05; ^†^Mann-Whitney test and ^‡^Wilcoxon signed-rank test were applied for non-parametric data

## Discussion

Multifocal IOLs provide multiple focal points to correct vision at various distances but are not recommended for patients with glaucoma because of reduced contrast sensitivity and photic phenomena^28^. Recently developed enhanced monofocal IOLs have demonstrated improvements in intermediate vision and have been found to be effective even in patients with early glaucoma^24,29–31^. However, there is a paucity of research on the use of enhanced monofocal IOLs in patients with advanced or severe glaucoma, in which reduced contrast sensitivity and VF restrictions are significant concerns^12,14^. Therefore, we aimed to evaluate the safety and efficacy of enhanced monofocal IOLs in patients with varying degrees of glaucoma, particularly severe cases.

There were significant baseline differences between the enhanced monofocal and standard monofocal IOL groups, particularly in age and the composition of glaucoma types. These differences likely reflect clinical practices where older patients and those with more severe glaucoma are more often recommended standard monofocal IOLs due to their cost-effectiveness and established safety profiles. Recognizing these differences, we analyzed changes in visual outcomes rather than absolute postoperative values to better isolate the impact of IOL type. This approach allowed us to account for baseline differences and better assess the true efficacy of enhanced monofocal IOLs relative to that of standard IOLs. Nevertheless, it is important to consider that the more advanced glaucoma in the standard IOL group may inherently limit the magnitude of postoperative improvements in visual function, potentially influencing the observed results.

Both IOL types significantly improved BCVA, MD, and RNFLT after cataract surgery. Analysis by glaucoma type yielded similar results, except for the RNFLT in eyes with PACG. This indicates that cataract surgery using either type of IOL significantly enhances visual function in patients with glaucoma^32^. Importantly, the magnitude of these improvements was comparable between the two groups, implying that enhanced monofocal IOLs are as effective as standard monofocal IOLs in improving visual outcomes. Postoperative IOP reductions, which were comparable between the two groups, further underscore the safety of both IOL types.

After verifying the overall safety and efficacy of the enhanced monofocal IOL, we evaluated the results according to glaucoma severity. Next, we analyzed whether there were differences based on the presence or degree of central VF defects within 10°. With the progression of glaucoma, the degree of central VF involvement advances, and as macular function deteriorates, contrast sensitivity decreases^12–14^. Central VF defects are often observed in early glaucoma^33,34^, and even relatively small initial defects can diminish the vision-related quality of life^35–37^. Additionally, patients with similar MD values in VF tests may exhibit different patterns of VF defect. Therefore, to accurately assess the use of enhanced monofocal IOLs in patients with glaucoma, it is important to perform evaluation not only on the basis of severity, but also with respect to the presence and extent of central VF damage.

In all patients, BCVA and RNFLT increased significantly after surgery, irrespective of glaucoma severity or the presence and extent of central VF defects. Additionally, no significant difference was observed between the two types of IOLs. In the VF test results, including VFI and MD, there were no significant differences in the changes before and after surgery between the two types of IOLs. The lack of difference between the two IOL types in the VF tests suggests that it may also be safe to evaluate glaucoma after surgery. These findings align with recent studies demonstrating that enhanced monofocal IOLs provide significant visual improvements without compromising retinal sensitivity, even in glaucoma patients^38^.

A significant difference in IOP changes was observed between the two groups in N 5–8, while no significant differences were found in N 1–4 or N 9–12. Importantly, none of the groups experienced postoperative IOP elevation, alleviating concerns regarding potential glaucoma progression following cataract surgery.

However, the present study did not include specific evaluations such as defocus curves and contrast sensitivity testing. Incorporating these evaluations in future studies could provide a more comprehensive understanding of the advantages of enhanced monofocal IOLs.

Various types of IOL have been developed considering lifestyle changes such as increased smartphone usage and the corresponding rise in the demand for near and intermediate vision. However, patients with glaucoma, especially those in the severe or advanced stages, have not been considered ideal candidates for these premium IOLs. It was disappointing for glaucoma specialists that, despite the development of enhanced monofocal IOLs designed to overcome the drawbacks of multifocal IOL^21,22^, which have shown favorable results in patients with early glaucoma^24^, research on their application in severe cases is lacking. This limitation restricts the available options for IOL selection.

From this perspective, the fact that the enhanced monofocal IOLs showed results comparable to the standard monofocal IOLs, irrespective of glaucoma severity or the extent of central VF defects, is highly noteworthy. Patients with glaucoma can choose better-performing IOLs that suit their lifestyles.

This study had some limitations. First, we could not analyze intermediate VA and contrast sensitivity due to the lack of information. These factors can substantially affect subjective satisfaction and QOL, particularly in patients with advanced or severe glaucoma whose QOL is already diminished. Therefore, additional prospective studies on these aspects are necessary. Second, the retrospective, comparative, case series design of this study precluded randomization, such as potential differences in IOL preferences. However, considering the absence of studies on the use of enhanced monofocal IOLs in severe or advanced glaucoma, our study provides valuable real-world insights. Further, to minimize biases in the IOL selection process, we compared the pre-operative values across different IOL types to ensure that there were no significant differences that could distort the results (Supplementary Material 2). Third, the sample size for severe glaucoma or significant central VF loss was relatively small. Since the important results of this study are negative findings, indicating no significant difference between the two IOL types, caution should be exercised while interpreting them. We expect to gather more data as the use of enhanced monofocal IOL in our hospital increases. Finally, the post-surgical follow-up period was insufficient. Glaucoma is a progressively advancing disease, and since enhanced monofocal IOLs are preferred in younger patients, a comparison of long-term outcomes is necessary.

Despite the above-mentioned limitations, the most significant finding of this study was that enhanced monofocal IOLs elicited comparable results to standard monofocal IOLs, irrespective of the severity of glaucoma and the extent of central VF defects.

In conclusion, enhanced monofocal IOLs demonstrated significant efficacy and safety in improving visual outcomes in patients with glaucoma of varying severities. They offer a promising alternative to standard monofocal IOLs, potentially providing better intermediate vision, while maintaining overall visual function. This study supports their broader use in the management of cataracts in patients with glaucoma, contributing to more informed IOL selection and improved patient care.

## Electronic supplementary material

Below is the link to the electronic supplementary material.

Supplementary Material 1

Supplementary Material 2

## Data Availability

The datasets generated and analyzed in the current study are available from the corresponding author upon reasonable request.

## Glossary

BCVA (Best-Corrected Visual Acuity): The best possible vision a person can achieve with corrective lenses, measured in terms of visual acuity.
MD (Mean Deviation): A statistical measure used in visual field testing to represent the average deviation of the test results from the normal reference values.
RNFLT (Retinal Nerve Fiber Layer Thickness): The thickness of the layer of nerve fibers in the retina, an important indicator in glaucoma assessment.
VFI (Visual Field Index): A global index that estimates the overall field loss in a visual field test.
