# Supplementary material for: Efficacy and safety of the enhanced monofocal intraocular lens in glaucoma of varying severity
Source: Sci Rep. 2025 Feb 8;15:4737. doi: 10.1038/s41598-025-87282-3 (PMC11807180; doi:10.1038/s41598-025-87282-3)
Supplement: Supplementary file 1 — Supplementary Material 1 [file 41598_2025_87282_MOESM1_ESM.docx]

**Supplementary Material 1. Definition of Significant Points by SCHEIE grading system**

| **Definition of Significant Points** | |
| --- | --- |
| **Point value** | **Rule** |
| < 0.5% | One point counts. |
| < 1% | One point counts. |
| < 2% | A point only counts if it is next to a <2%, <1%, or <0.5% point in the same hemifield. The points may be in contact diagonally. |
| < 5% | This point never counts for itself and never causes a <2% point to count. |
|  | |
