# Supplementary material for: Efficacy and safety of the enhanced monofocal intraocular lens in glaucoma of varying severity
Source: Sci Rep. 2025 Feb 8;15:4737. doi: 10.1038/s41598-025-87282-3 (PMC11807180; doi:10.1038/s41598-025-87282-3)
Supplement: Supplementary file 2 — Supplementary Material 2 [file 41598_2025_87282_MOESM2_ESM.docx]

**Supplementary Material 2. Comparison of preoperative values between the two IOL groups**

| **Parameter** | **Enhanced monofocal IOL** | **Standard monofocal IOL** |  |
| --- | --- | --- | --- |
| **By Severity** |  |  | **p-value** |
| **Early** | **n = 88** | **n = 65** |  |
| Age (years) | 64.55±8.99 | 69.02±7.35 | 0.001* |
| Baseline visual indices |  |  |  |
| Pre-operative BCVA (logMAR) | 0.37±0.38 | 0.35±0.28 | 0.841 |
| Pre-operative VFI (%) | 95.48±3.65 | 94.85±5.20 | 0.404 |
| Pre-operative MD (dB) | -2.84±2.03 | -3.25±1.64 | 0.177 |
| Pre-operative RNFL thickness (μm) | 83.42±13.27 | 82.15±15.04 | 0.589 |
| **Moderate** | **n = 40** | **n = 30** |  |
| Age (years) | 70.03±7.34 | 66.57±8.19 | 0.077^†^ |
| Baseline visual indices |  |  |  |
| Pre-operative BCVA (logMAR) | 0.52±0.46 | 0.55±0.51 | 0.853^†^ |
| Pre-operative VFI (%) | 83.00±8.92 | 85.43±9.21 | 0.187^†^ |
| Pre-operative MD (dB) | -8.22±1.81 | -8.37±1.87 | 0.812^†^ |
| Pre-operative RNFL thickness (μm) | 74.30±14.58 | 78.53±14.05 | 0.072^†^ |
| **Severe** | **n = 28** | **n = 45** |  |
| Age (years) | 65.29±10.90 | 68.47±10.02 | 0.289^†^ |
| Baseline visual indices |  |  |  |
| Pre-operative BCVA (logMAR) | 0.54±0.62 | 0.73±0.61 | 0.046^†^* |
| Pre-operative VFI (%) | 37.21±26.75 | 38.84±20.93 | 0.807^†^ |
| Pre-operative MD (dB) | -22.19±7.02 | -21.00±5.51 | 0.606^†^ |
| Pre-operative RNFL thickness (μm) | 66.00±19.40 | 60.70±17.16 | 0.089^†^ |
| **By Central visual field defect** |  |  | **p-value** |
| **N 1-4** | **n = 58** | **n = 34** |  |
| Age (years) | 67.07±9.28 | 69.85±8.13 | 0.159^†^ |
| Baseline visual indices |  |  |  |
| Pre-operative BCVA (logMAR) | 0.35±0.35 | 0.47±0.44 | 0.139^†^ |
| Pre-operative VFI (%) | 87.59±10.01 | 90.24±9.04 | 0.124^†^ |
| Pre-operative MD (dB) | -5.91±4.32 | -5.84±4.40 | 0.799^†^ |
| Pre-operative RNFL thickness (μm) | 78.88±15.36 | 76.09±15.76 | 0.761^†^ |
| **Parameter** | **Enhanced monofocal IOL** | **Standard monofocal IOL** | **p-value** |
| **N-5-8** | **n = 22** | **n = 18** |  |
| Age (years) | 68.68±8.51 | 69.85±8.13 | 0.201^†^ |
| Baseline visual indices |  |  |  |
| Pre-operative BCVA (logMAR) | 0.47±0.41 | 0.54±0.49 | 0.671^†^ |
| Pre-operative VFI (%) | 71.36±13.88 | 61.28±14.56 | 0.045^†^* |
| Pre-operative MD (dB) | -11.10±4.81 | -13.95±4.75 | 0.079^†^ |
| Pre-operative RNFL thickness (μm) | 72.14±12.53 | 71.00±16.89 | 0.693^†^ |
| **N 9-12** | **n = 17** | **n = 32** |  |
| Age (years) | 63.76±12.04 | 67.47±9.13 | 0.406^†^ |
| Baseline visual indices |  |  |  |
| Pre-operative BCVA (logMAR) | 0.62±0.71 | 0.83±0.65 | 0.123^†^ |
| Pre-operative VFI (%) | 24.29±27.36 | 32.84±21.72 | 0.089^†^ |
| Pre-operative MD (dB) | -24.90±8.83 | -22.19±6.60 | 0.078^†^ |
| Pre-operative RNFL thickness (μm) | 62.76±20.31 | 58.16±16.19 | 0.178^†^ |

BCVA, best-corrected visual acuity; IOL, intraocular lens; VFI, visual field index; MD, mean deviation; RNFL, radiating nerve fiber layer; IOP, intraocular pressure; logMAR, logarithm of the minimum angle of resolution

*p<0.05; ^†^Mann-Whitney U test
